# Costimulatory Pathways: Physiology and Potential Therapeutic Manipulation in Systemic Lupus Erythematosus

**DOI:** 10.1155/2013/245928

**Published:** 2013-07-29

**Authors:** Nien Yee Kow, Anselm Mak

**Affiliations:** Division of Rheumatology, Department of Medicine, University Medicine Cluster, Yong Loo Lin School of Medicine, National University of Singapore, Level 10, NUHS Tower Block, 1E Kent Ridge Road, Singapore 119228

## Abstract

System lupus erythematosus (SLE) is an immune-complex-mediated autoimmune condition with protean immunological and clinical manifestation. While SLE has classically been advocated as a B-cell or T-cell disease, it is unlikely that a particular cell type is more pathologically predominant than the others. Indeed, SLE is characterized by an orchestrated interplay amongst different types of immunopathologically important cells participating in both innate and adaptive immunity including the dendritic cells, macrophages, neutrophils and lymphocytes, as well as traditional nonimmune cells such as endothelial, epithelial, and renal tubular cells. Amongst the antigen-presenting cells and lymphocytes, and between lymphocytes, the costimulatory pathways which involve mutual exchange of information and signalling play an essential role in initiating, perpetuating, and, eventually, attenuating the proinflammatory immune response. In this review, advances in the knowledge of established costimulatory pathways such as CD28/CTLA-4-CD80/86, ICOS-B7RP1, CD70-CD27, OX40-OX40L, and CD137-CD137L as well as their potential roles involved in the pathophysiology of SLE will be discussed. Attempts to target these costimulatory pathways therapeutically will pave more potential treatment avenues for patients with SLE. Preliminary laboratory and clinical evidence of the potential therapeutic value of manipulating these costimulatory pathways in SLE will also be discussed in this review.

## 1. Introduction

 Systemic lupus erythematosus (SLE) is an autoimmune condition in which autoantibodies against various nuclear and nonnuclear antigens trigger immune-complex-mediated inflammation that can result in organ damage [1]. Similar to other inflammatory and autoimmune conditions, the pathogenic processes that contributed to SLE involve complex interactions between immunocytes and nonimmunocytes such as endothelial and epithelial cells through a number of receptor-ligand systems [2]. Such receptor-ligand association is crucial in initiating and regulating both innate and adapted immunity, from initial presentation of danger signals to naïve immunocytes by professional antigen-presenting cells (APCs) to the moment when communication between T and B cells is driven by specific antigenic epitopes. The cognate T-B-cell interactions which lead to T-cell-dependent autoantibody production by the B cells are central to the pathogenesis of SLE [3]. 

 Generally, antigen presentation is broadly divided into four stages: adhesion, antigen-specific activation, costimulation, and cytokine production and signalling [4]. Instead of initiating cell activation, the process of adhesion which occurs between cells, as well as between cells and extracellular matrix that requires adhesion molecules such as integrins and selectins, is essential in bridging the cells together before subsequent cellular activation takes place [5]. After physically approximating immunologically important APCs and lymphocytes, the APCs capture, internally process, and present peptides of antigen-specific sequences to T lymphocytes through interaction between the major histocompatibility complex (MHC) of the APCs and T-cell receptors (TCRs) on the T cells in an antigen-specific manner. The binding and interaction between the MHC and TCR are, however, insufficient to propagate and sustain downstream proinflammatory signals. Additional intercellular signals in the form of costimulatory pathways are required to perpetuate the ensuing proinflammatory pathways [6]. At this stage, communication between the cells involved through costimulatory signals is critical in determining if such cell-cell association is ensues and terminates. Indeed, besides perpetuating proinflammatory signals, costimulatory pathways are also crucial in attenuating the active immune system or generating peripheral immune tolerance for avoiding unnecessary tissue damage incurred by the overwhelmingly active proinflammatory effector signals [7]. In the subsequent sections, a brief overview of the physiology of well-characterized costimulatory molecules will be highlighted, followed by a discussion of the relevance of some pathophysiologically important costimulatory pathways operant in SLE. Emerging data regarding the therapeutic potential of costimulatory molecule blockades on laboratory benches and randomized controlled clinical trials will also be discussed. 

## 2. An Overview of Costimulatory Molecules and Their Signalling Pathways

### 2.1. CD80/86-CD28 and ICOS-B7RP1

 Costimulatory molecules are broadly divided into those belong to the immunoglobulin (Ig) superfamily (IgSF) and the tumour necrosis factor (TNF) superfamily (TNFSF) based on their phenotypic and signalling features. The CD28 and CD80 (B7-1)/CD86(B7-2) represent one of the most relevant costimulatory pathways of the Ig family. During the very early stage of T-cell activation, CD28 is expressed on T cells and is ligated by CD80/CD86 which are constitutively expressed on dendritic cells (DCs) and inducible in other professional APCs such as B cells and monocytes. CD28 stimulation has been shown to prolong and augment IL-2 production from T cells and prevent the development of peripheral immune tolerance [8]. Consequently, the stimulated T cells further mature, differentiate, and signal the B cells to proliferate and differentiate into antibody-producing plasma cells for production of antibodies. More costimulatory pathways are further activated subsequently to enhance the cognate interactions between APCs and T lymphocytes and intensify the proinflammatory effector signals.

Another recently identified costimulatory system of the same group as CD28-CD80/86 is the inducible costimulator (ICOS)-B7-related protein 1 (B7RP1) pathway. The ICOS which shares similar structural and functional similarities as CD28 was characterized in 1999 [9, 10]. Structurally, ICOS is similar to CD28 in that it is also a transmembrane glycoprotein which is expressed on activated T cells. Functionally, on binding with its ligand B7RP1 which is constitutively expressed on B cells and inducible on monocytes and DC [9, 10], ICOS triggers germinal centre formulation, clonal expansion of T cell, T-cell-dependent antibody production, and isotype switching of B cells [10]. ICOS has been found to be highly expressed on activated CD4+ T cells in patients with autoimmune conditions such as rheumatoid arthritis (RA), SLE, and inflammatory bowel disease [11–13]. On the other hand, ICOS deficiency is found in patients with primary immunodeficiency, with impaired CD4 and CD8 effector functions and reduced memory T-cell population, together with features of common variable immunodeficiency characterized by hypogammaglobulinemia and recurrent bacterial infection [14]. 

### 2.2. CD70-CD27

After initial activation of T cells is achieved via MHC-TCR and CD80/86-CD28 interactions, CD70 (TNFSF7) starts to be expressed on the activated T cells and its cognate association with CD27, which is expressed on B and natural killer (NK) cells, serves to strengthen the costimulatory signals between the immunocytes [15]. Activation of CD70 enhances T-cell-dependent antibody production by promoting germinal center formation, B-cell activation, and clonal expansion, as well as differentiation into plasma cells [16]. In the T-cell compartment, cognate interaction between CD70 and CD27 also induces the proliferation of and cytokine secretion by CD4+ and CD8+ T cells and development of cytotoxic T-cell responses by CD8+ T cells [17]. 

### 2.3. CD40-CD40L

CD40 belongs to the TNFSF (TNFSF5) and is expressed on APCs, B cells, and traditional nonimmune cells such as endothelial, epithelial, and renal tubular cells [18]. On activation upon ligation with CD40L expressed on activated T cells, CD40 can deliver even stronger activation signals than that of surface bound Ig to B cells. Activated T cells transiently express the CD40L which interacts with CD40 on B cells and further drives B-cell differentiation, maturation, and isotype switching, especially those in the germinal centers [19]. In addition, signal transduction through CD40 upregulates the expression of CD80/CD86 which further intensifies costimulatory signals to the T cells involved in the interaction [20]. Overexpression of CD40L on T cells has been found in a number of autoimmune diseases which are characterized by overproduction of autoantibodies [21, 22]. On the other hand, deficiency of CD40L, such as in patients with CD40 gene mutation, would lead to Ig class-switch recombination deficiency characterized by high IgM but low IgA and IgG levels and recurrent opportunistic infection [23]. 

During the later stage of T-cell activation, other costimulatory molecules which belong to the TNFRSF such as CD137 and OX40 start to participate in the scene of costimulation. These molecules are only expressed on the activated T cells during the later stage of activation. Expressions of these costimulation molecules are required for further sustaining differentiation of the activated T cells, with an aim to prolong their survival and perform specific effector and memory functions [24]. 

### 2.4. CD137-CD137L

CD137 belongs to TNFSF9, and it is a potent costimulatory receptor molecule which is mainly expressed on stimulated T cells and NKT cells [25]. In DCs and CD4+ CD25+ Foxp3+ Treg cells, CD137 is expressed in a constitutive manner and around 48 hours after initial stimulation, CD137 is predominantly expressed on CD4+ and CD8+ T cells [26, 27]. In both murine system and humans, cognate interaction between CD137 and CD137L on activated T cells and B cells, respectively, leads to proliferation and differentiation of both interacting cells and results in substantial Ig production, before apoptosis of B cells or APCs that occurs upon prolonged stimulation at the terminal stage of immune activation [28]. Indeed, bidirectional signalling ensues in the T and B cells or APCs during CD137-CD137L interaction. Such mutual signalling leads to initial stimulation of both communicating cells followed by their apoptosis, suggesting that CD137 ligation acts as an initial checkpoint to attenuate the over-active proinflammatory effector response [28]. 

### 2.5. OX40-OX40L

 OX40 (CD134) belongs to the TNFSF4. Similar to CD137, OX40 is expressed on activated CD45RO+ CD4+ T lymphocytes [29]. Signalling of OX40 requires and is controlled by its ligand, OX40L (CD134L), which is constitutively expressed on APCs including B cells, macrophages, DC, and endothelial cells [30]. While OX40 signalling enhances survival of T cells and their subsequent cytokine production and expansion of their memory cell pool, stimulation of OX40L enhances B-cell proliferation and differentiation [31]. A recent study has found that OX40L stimulation inhibited the generation of IL-10-producing CD4+ Tregs cells from memory and naive T cells. In addition, OX40L ligation inhibited IL-10 production and suppressive function of differentiated type 1 regulatory CD4+ T cells (Tr1) [32]. 

### 2.6. Downregulation of Proinflammatory Signals during the Late Stage of T-Cell Activation

Costimulation cannot continue unchecked or else overwhelming proinflammatory response will ultimately ruin the host. The costimulatory system consists of peculiar mechanisms which can dampen the proinflammatory signals, achieve immune homeostasis, and assist in the development of peripheral tolerance. CTLA-4 (CD152), a homolog of CD28, starts to be expressed on activated T cell and binds to CD80/86 when T-cell activation has reached its peak [33]. Indeed, the affinity between CTLA-4 and CD80/86 is much higher than that between CD28 and CD80/86 [34, 35]. CTLA-4 activation signals the T cells to attenuate its proliferation and proinflammatory effector signals [36]. Once the CTLA-4 is upregulated, expression of CD28 on T cells will start to be downregulated by endocytosis [36]. Apart from CTLA-4, prolonged interaction between CD137 and CD137L leads to apoptosis of the participating cells which also results in attenuation of the proinflammatory signals [28]. One of the mechanisms postulated is that prolonged activation through CD137 signalling induces large amount of IFN*γ* production by CD8+ T cells, which in turn stimulates apoptosis of B cells and DCs and results in downregulation of Ig production and CD80/86 expression respectively. Downregulated B7 expression in DCs further weakens the communication with T cells and attenuates the costimulatory signals [28]. 

### 2.7. Intracellular Signalling Pathways of Costimulatory Molecules

**Figure 1:**
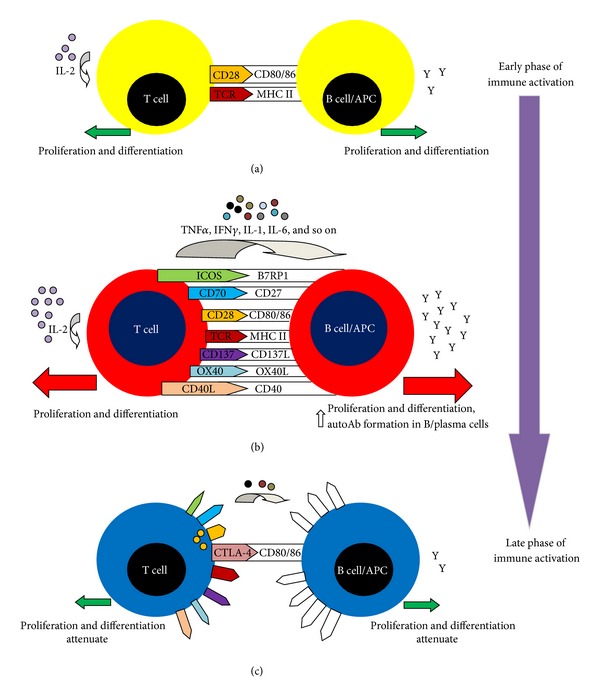
Expression of costimulation molecules in different stages of immune activation and their concerted effector functions. (a) Initial phase of immune activation; (b) maximal phase of immune activation with expression of many costimulatory molecules (figure is for depiction only, costimulation molecules may not be all expressed in a single immune reaction), coupled with proliferation and differentiation of immunocytes, production of proinflammatory cytokines and autoantibodies; (c) after reaching the peak of activation, CTLA4 expressed on T cells interacts with CD80/86 on B cells/APCs with higher affinity than CD28. Immune response attenuates and costimulatory pathways start to disintegrate. CD28 molecules expressed during maximal activation phase are endocytosed, pictorially depicted as intracellular small round yellow vesicles in the T cells of condition (c). Abbreviations: TCR: T-cell receptor; MHC II: class II major histocompatibility complex; APC: antigen-presenting cells; and autoAb: autoantibodies.

 While a number of downstream signalling pathways after stimulation of costimulatory molecules have been discovered, the functions of many of these pathways are still not fully understood, not mentioning that there are many signalling pathways which have not been discovered or functionally characterized. Nevertheless, Table 1 summarizes the signal transduction pathways of costimulatory molecules in which their functions have been relatively well characterized. Classification of these costimulatory molecules, cells which they are expressed on, and their physiological functions are also briefly described in Table 1.  Figure 1 depicts the expression of costimulatory molecules and their concerted effector actions in relation to different stages of immune activation. 

## 3. Costimulatory Molecules and SLE

### 3.1. CD80/86-CD28 Interaction

 Involved in the early stage of T-cell activation, the interaction between CD80/86 and CD28 has been extensively studied amongst various costimulatory molecules in SLE. A number of animal studies attempting to block the CD80/86-CD28 system revealed promising results in terms of amelioration of lupus-related glomerulonephritis, autoantibody production, and class switching, as well as mortality [37, 38]. In a murine lupus model, it has been shown that CTLA4-Ig, a recombinant fusion protein comprising a Fc fragment of human IgG1 which binds either to CD80 or CD86 with a much higher avidity than CD28, contracted the autoreactive B-cell population and reduced autoantibody production and Ig class switching [39]. In clinical studies, CTLA-4Ig (abatacept) has been shown to be able to dampen the interaction between T and B lymphocytes, leading to potential amelioration of autoimmune-driven inflammation [40]. While promising efficacy and safety of abatacept have been demonstrated for the treatment of RA [41], the results of two-phase IIb/III clinical trials testing abatacept in SLE are rather disappointing [42, 43]. In a one-year multicentre, double-blind, placebo-controlled trial of 175 patients with moderately active lupus presenting with arthritis, serositis, and discoid lupus receiving either abatacept (10 mg/kg) or placebo, no statistically significant difference was found between the 2 groups in the primary (new BILAG A or B flare) and the secondary study endpoints [42]. Nevertheless, in *post hoc* analyses while only BILAG A flare was taken into account, significantly more patients receiving placebo experienced flares (54.4%) than those who received abatacept (40.7%). In addition, greatest benefit was seen in patients with arthritis, and improvement of patient-reported outcomes such as sleep disturbance, health-related quality of life, and fatigue was more substantial in the abatacept-treated group [42]. Although more serious adverse events were reported in the abatacept-treated group (19.8% versus 6.8%), *post hoc* analyses did provide evidence that abatacept may confer beneficial effect in severe nonrenal lupus and moderate lupus manifestations [42]. 

 A more recent 52-week phase III clinical trial testing abatacept for its safety and efficacy in 290 patients with ISN/RPS class III or IV lupus nephritis on a background of mycophenolate mofetil and glucocorticoids was published in abstract form [43]. Again disappointingly, no significant difference was found between the treatment and placebo groups in time to achieve complete renal response. However, as in the nonrenal lupus trial [42], *post hoc* analysis revealed that a modified complete response was higher in patients who received abatacept [43]. Repeated analyses of these data by less stringent criteria such as the ALMS and LUNAR response criteria also revealed greater difference in complete response favouring the abatacept-treated groups [44]. A randomized controlled phase II trial (ACCESS) comparing the combination therapy of cyclophosphamide (500 mg intravenous infusion fortnightly for 12 weeks) and abatacept (500–1000 mg at 0, 2, and 4 weeks then monthly for 24 to 48 weeks) versus standard cyclophosphamide (500 mg intravenously infusion fortnightly for 12 weeks) in active lupus nephritis is currently underway [45]. 

 Blockade of CD80 by monoclonal antibody has recently been explored in the treatment of lupus-like disease in a mouse model. In a pristane-induced lupus-like disease mouse model, monoclonal antibody against CD80 was shown to be able to attenuate inflammatory response and severity of lupus-like signs [46]. 

### 3.2. CD70/CD27 Interaction

Before discussing the impact of the CD70-CD27 interaction on the pathogenesis of SLE, it is useful to highlight the relationship between DNA methylation and SLE. In general, DNA methylation involves the addition of a methyl group to adenosine or cytosine base of the DNA nucleotides and stably alters gene expression [47]. In the early 1990s, global reduction of DNA methylation by 15–20% was first identified in lupus T cells [48]. Subsequently, the link between DNA hypomethylation, T-cell autoreactivity, and development of SLE was further supported by the association between low 5-methylcytosine content of DNA in blood and disease activity in patients with SLE [49]. Thus, methylation status of lupus susceptible genes such as those coding CD70 and CD40L may affect the clinical expression of SLE. In an animal model of lupus, CD70 overexpression on splenic CD4+ cells was observed in 16-week-old MRL/lpr mice with established lupus-like disease but not in their 5-week-old counterparts prior to disease development. Additionally, CD70 expression was found to correlate with DNA hypomethylation of the CD70 gene in activated CD4+ T cells [50]. The expression of DNA methyltransferase 1 (DNMT1) was found to be significantly lower in the 16-week-old mice than that of the 5-week-old mice, and the low DNMT1 level led to T-cell DNA hypomethylation, CD70 overexpression, and consequently age-dependent development of lupus-like disease in the MRL/lpr mice [50].

In humans, CD4+ T cells of patients with SLE were also shown to overexpress CD70 when compared to healthy subjects, although CD70 expression on CD4+ T cells has not been found to be consistently correlated with lupus disease activity [51–53]. Nevertheless, overexpression on T cells in SLE patients was confirmed to be the result of hypomethylation of DNA sequences that flank the CD70 promoter due to reduced expression of DNMT1, which results in failure in downregulating CD70 expression once it is induced by T-cell activation [54]. In a recent study which aimed to unravel the mechanism of DNA hypomethylation of T lymphocytes in patients with SLE, a transcription factor named regulatory factor X1 (RFX1), which functions to recruit DNMT1 to the promoter region of CD70, was shown to be downregulated in human lupus CD4+ T cells, potentially contributing to CD70 overexpression [55]. CD70 overexpression in SLE has been postulated to intensify B cell costimulation with subsequent increase in autoreactive Ig production [52]. In addition to hypomethylation, post-transcriptional modifications on histone protein also play a role in overexpressing CD70 in lupus T cells [56]. *In vitro* treatment of lupus CD4+ T cells with a histone deacetylase inhibitor was demonstrated to overexpress CD70 in these cells by aberrant histone modifications (increase in H3 and H4 acetylation levels) within the TNFSF7 promotor region [56]. 

High expressions of CD27 in B cells and plasma cells were shown to correlate with SLE disease activity in terms of higher SLEDAI, anti-dsDNA IgG levels, and lower serum complement levels in a cross-sectional study of 59 patients with SLE [57]. However, concurrent infection in these patients with elevated CD27 expression in their plasma cells limits the clinical utility of CD27+ plasma cell level as an ideal marker of lupus disease activity [57]. 

### 3.3. CD40-CD40L Interaction

 The CD40L gene, similar to that of CD70, is methylation sensitive. The CD40L gene is a SLE susceptible gene located on the X chromosome. Partly as a consequence of demethylated regulatory sequence on the inactive X chromosome, CD40L was found to be overexpressed on T cells of female lupus patients [58, 59]. CD4+ T cells from female lupus patients treated *in vitro* with DNA methylation inhibitors were shown to overexpress CD40L mRNA, and elevation of CD40L expression has been unanimously shown to promote autoantibody production in B cells in the presence of their strong interaction with CD40 expressed on T cells [58, 59]. Blocking the interaction between CD40L and CD40 is thus a feasible approach to ameliorate autoantibody production and hence the clinical manifestation of SLE [59]. In fact, CD40L blockade has been shown to prevent the development of SLE effectively in a lupus mouse model [60].

 Clinical trials testing the blockade of the CD40-CD40L pathway in the treatment of SLE are disappointing [61, 62]. In addition to the failure of meeting the predefined study end-points, the unfavourable side-effect profile of anti-CD40L (BG9588) led to premature termination of a multicentre phase II trial of BG9588 in SLE [62]. In a double-blind, placebo-controlled, multiple-dose study, 85 patients with mild to moderately active SLE were randomized to receive 6 infusions of IDEC-151 of 2.5, 5, and 10 mg/kg and placebo at 0, 2, 4, 8, 12, and 16 weeks [61]. At 20 weeks, although the SLEDAI scores improved in all groups from baseline, statistical significance was not reached amongst the different groups. No difference in the predefined secondary outcomes including BILAG, physician and patient global assessment, Krupp fatigue assessment, serum anti-dsDNA and complement levels, and quality of life was noted. Adverse events were comparable between both groups, and none of the subjects developed anti-CD154 antibody after 16 weeks of treatment [61]. In another smaller phase II, open-label trial of evaluating anti-CD40L antibody (BG9588) in the treatment of 28 patients with proliferative lupus glomerulonephritis (20 mg/kg biweekly for 3 doses then monthly for 4 additional doses), the occurrence of 2 myocardial infarctions 59 and 9 days after infusion, respectively, led to premature termination of the trial, even though patients on BG9588 treatment demonstrated significant reduction of proteinuria, haematuria, and anti-dsDNA titre with increase in serum C3 levels [62]. 

### 3.4. CD137-CD137L Interaction

Although CD137 activation upon ligation with CD137L provokes bidirectional signalling which induces proliferation and differentiation of both T and B cells, knocking out CD137 paradoxically induces more autoantibody production, higher level of pathogenic CD4+ and double negative T cells (CD3+CD4-CD8-B220+), more severe cutaneous lupus, glomerulonephritis, and death in the MRL/lpr mouse model [63]. The results led to the hypothesis that if CD137 is agonized, the opposite effects which favour amelioration of SLE might occur. Two separate studies using knockout mice of different backgrounds unanimously demonstrated amelioration of lupus phenotypically and serologically by agonizing CD137 receptor [64, 65]. By injecting agonistic CD137 monoclonal antibody (mAb), reduction in glomerulonephritis in MRL/lpr mice was demonstrated, besides reduction in CD4+ lymphocytes, anti-dsDNA production, germinal centre formation in secondary lymphoid organs, and mortality [65]. The same mAb injected into NZWB/W lupus-prone mice in another study produced similar results, except that no reduction of CD4+ was demonstrated [64]. Instead, an elevation of splenic CD25+ Treg cells was shown in the agonistic CD137 mAb group when compared with injection of isotype control [64]. Of particular note is that while agonizing CD137 has shown benefits in reducing the pathogenic autoantibodies and lymphocytes and amelioration of clinical manifestation of SLE in animal models, a number of *in vitro* studies using nonlupus human samples have revealed that CD137 activation enhances ingression of monocytes and their interaction with ICAM-1 in blood vessels, leading to atherosclerosis and plaque inflammation which may potentially amplify cardiovascular risk [66–68]. 

### 3.5. OX40/OX40L

 OX40 (CD134) expression in lupus PBMCs is predominantly restricted in CD3+ CD4+ CD45RO+ T lymphocytes, and the level correlates with lupus disease activity [69]. OX40 has also been found to be highly expressed in kidneys of patients with lupus nephritis [70]. While there have been no therapeutic trials performed in animals and humans addressing the safety and efficacy of blocking OX40-OX40L in the treatment of SLE, *in vitro* studies revealed that anti-CD134mAb-treated splenocytes of BXSB mice expressed significantly less markers of active SLE such as anti-dsDNA, IL-6, and IFN*γ* [71]. In a small mechanistic study of 10 patients with SLE, stimulation of PBMCs under the influence of anti-CD134 mAb resulted in reduction of Th2 cytokine but increase in IFN*γ* production [72]. Stimulation with CD134-Fc fusion protein further reduced the production of both Th1 and Th2 cytokines including IL-4, IL-10, and IFN*γ* in patients with lupus nephritis [72], suggesting the potential role of anti-CD134 in reducing IL-4- and IL-10-mediated renal inflammation. In another study, expression of OX40 on IL-17-producing T cells was higher in SLE patients when compared with that of healthy controls [73]. Furthermore, expression of OX40 on T cells was correlated with lupus disease activity, and OX40+ T cells were found in renal biopsies of patients with lupus nephritis, which may signify that OX40+ T cells migrate to the nephritic kidneys and contribute to inflammation and IL-17 production by interaction with CD137L-expressing resident renal cells [73]. Culture of lupus PBMCs with CD134 mAb in another experiment demonstrated inhibition of haemolytic activity and perforin expression on the PBMCs, and the degree of inhibition was associated with the disease activity of SLE and the degree of proteinuria [74]. 

### 3.6. ICOS-B7RP-1

Without interaction with B7RP1, ICOS-deficient mice were noted to have reduced total IgG and anti-dsDNA production [75]. In NZB/W F1 mouse model, blockade of B7RP1 with anti-B7h mAb before the onset of renal disease significantly delayed the onset of proteinuria, inhibited all subclasses of IgG autoantibody production, reduced the degree of glomerulonephritis, and prolonged the survival of the mice [76]. Even after the appearance of proteinuria in established disease, anti-B7h mAb consistently improved the renal histopathology and disease progression in the animals [76]. Interestingly, in contrast to anti-B7h mAb treatment, anti-ICOS mAb treatment did not affect lymphocyte count and phenotypes of the animals. Instead, anti-ICOS mAb treatment of mice with severe combined immunodeficiency induced antigen-specific T-cell activation which led to the production of large amount of IFN*γ* and Th2 cytokines, more apoptosis, and cell death [76]. Based on these observations, monoclonal antibodies against ICOS functionally trigger ICOS signalling instead of inhibiting it. The timing and immunological milieu when ICOS signalling is manipulated are postulated to be crucial in determining the effect of ICOS blockade. For example, delayed but not early, ICOS blockade of a mouse cardiac allograft model was shown to enhance cardiac graft survival where CD28 costimulation was absent and while CD8+ T cells, CTLA-4, and the STAT-6 pathway were functionally active [77]. 

In humans, ICOS expression on CD4 and CD8+ T cells has been shown to be elevated in lupus patients, while ICOS-L (B7RP1) expression is downregulated in peripheral blood memory B cells after cognate interaction with ICOS+ T cells in coculture systems. In lupus kidney samples, it was found that ICOS+ T and B cells which infiltrated the kidneys were in close contact, suggesting that T-B interactions may also take place in peripheral tissues [12]. A phase Ib trial assessing anti-B7RP1 mAb (AMG 557) in the treatment of stable lupus has just been completed, and results are eagerly awaited [78]. 

Table 2 summarizes the major translational studies and clinical trials manipulating various costimulatory pathways in the treatment of SLE. 

## 4. Conclusion

Costimulatory pathways initiate and perpetuate proinflammatory processes by sustaining cognate interaction between activated T cells and APCs which is central to the pathogenic process of SLE. Equally important, costimulatory pathways are capable of attenuating the ensuing proinflammatory response which is overwhelmed, as exemplified by the interaction between CTLA-4 and CD80/86 and prolonged CD137 stimulation.

While most studies involving murine lupus models have concluded the superior efficacy and safety of costimulatory blockade in treating lupus, most clinical trials which evaluated costimulatory blockers in SLE to date, such as CD154 and CTLA-4Ig, did not meet the predefined therapeutic endpoints. Nevertheless, reanalyses of these clinical trial data with the use of less stringent clinical response criteria and *post hoc* analyses may still advocate superior therapeutic value of these costimulatory blockades in moderate and severe lupus manifestations. Based on what we have learnt from the lessons of failure, there are 2 concomitant directions which we should proceed for reevaluating promising pharmacological agents and exploring potential biologic therapies for SLE. First, more realistic study design for clinical trials with less stringent and more practical endpoints can be employed to restudy medications such as abatacept and anti-CD154 mAb. Second, a number of existing background treatments of SLE such as glucocorticoids and cyclophosphamide would obscure the potential therapeutic effects of the biologics being evaluated in clinical trials. As such, more thorough understanding of the molecular aspects of the existing treatments and their potential impact on the signalling mechanisms of the medications under evaluation will be beneficial. While the current data from clinical trials testing the efficacy and safety of anti-CD154 and CTLA-4Ig for the treatment of SLE have not been promising, novel yet potentially safer targets based on our current knowledge need to be explored. For example, instead of targeting CD137, antagonizing CD137L may be of potential to reduce disease severity and cardiovascular complications because agonizing CD137 may induce atherosclerosis which is potentially detrimental to patients with SLE [66–68]. Similarly, antagonizing B7RP1 may be safer because targeting at ICOS may actually stimulate it and potentially triggers more severe autoimmunity [76].

## Figures and Tables

**Table 1:** Summary of the signalling mechanisms and physiological actions of major co-stimulatory molecules in systemic lupus erythematosus.

Costimulation receptor (ref.)	Costimulation ligand	Family	Cells expressing	Signaling molecules involved	Action
*CD80 *CD86 [79, 80]	CD28	IgSF	CD80/86: APCs including monocytes, M*ϕ*, and DC CD28: mainly naive CD4+ & CD8+ T cells during their initial phase of activation	CD28: phosphorylation by Lck and FYN, and ITK which recruits PI3K and Grb2 PI3K converts PI to PIP_3_ and activates Akt and subsequently NF-*κ*B Grb2 binds to SOS1, phosphorylates VAV1 which in turn activates Rac1 and JNK	CD80/86 are constitutively expressed on APCs and B cells CD80/86 are upregulated by inflammation and stimulation of T cells, and they provide costimulatory signals to CD28 and CTLA-4 Stimulation of CD28 prolongs and augments IL-2 production from T cells and prevents the development of peripheral immune tolerance
	CTLA-4	IgSF	T cells during late stage of activation	CTLA-4: phosphorylation by Lck, FYN, and RLK, binds to PI3K, SHP-2, and PP2A. PLC*γ*, and AKT, and hence proinflammatory effector signals are suppressed	Attenuates further costimulation between communicating immunocytes, dampens proinflammatory signals, and produces anergy. CTLA-4 expression induces CD28 endocytosis on activated T cells

B7RP1 [81]	ICOS	CD28-B7 family	ICOS: activated T cells B71RP1: APCs and B cells	ICOS: phosphorylation of ERK, JNK, and p38	ICOS stimulation induces further activation and clonal expansion of T cells, germinal centre formation, and T-cell- dependent antibody formation

CD137 [82, 83]	CD137L	TNFSF	CD137: activated T cells, NK-T cells CD137L: APCs, B cells	CD137L: Src tyrosine kinase which activates MEK1/2, P38 MAPK, subsequently, and NF-*κ*B (human). Association with TNFR1 which mediates reverse signalling	CD137 enhances proliferation of, and memory as well as cytolytic functions of T cells. It Inhibits CD4+ response and ameliorates autoimmunity due to IFN*γ* production by CD8+ T cells CD137L induces myelopoiesis, DC maturation, B-cell stimulation, and T-cell proliferation

CD27 [84]	**CD70	TNFSF	CD70: activated T and B cells and M*ϕ* CD27: resting T and NK cells, some in B cells	CD27 binds to TRAF 2/5 after trimerization and activates NF-*κ*Band the c-Jun pathway CD70 activates PI3K, Erk1/2, and MAPK	CD27 stimulation suppresses Th17 effector function and enhances B-cell activation and Ig production. CD70 signalling may regulate cell cycle of B cells and cytotoxicity of T cells

^†^OX40 [85, 86]	^‡^OX40L	TNFSF	OX40: T cells OX40L: APCs, glomerular endothelial cells	OX40L binds to OX40, recruits TRAF 2, 3, 5, and induces phosphorylation of I*κ*B*α*, and subsequently NF-*κ*B, PI-3K, and protein kinase B	OX40 signalling increases the longevity of T cells and subsequent cytokine production and expansion of memory T-cell population. OX40L signalling enhances B-cell proliferation and differentiation OX40L inhibits the generation of IL-10 producing CD4+ Tregs from naive and memory T cells

CD40 [87]	^€^CD40L	TNFSF	CD40: B cells CD40L: T cells	CD40: TRAF 1/2, 3/5, 5, 6, and induces NF-*κ*B while TRAF 2, 2/6, and 6 induces p38, Akt and JNK Jak 3 and induces STAT5 phosphorylation	Provides a strong activation signal to B cells for their differentiation, proliferation, and hence germinal centre development, and Ig response to T-dependent antigens. CD40 also upregulates CD80/86 expression and provides further stimulation signals to T cells.

Abbreviations: ref: references; CD: cluster of differentiation; IgSF: immunoglobulin superfamily; APCs: antigen-presenting cells; M*ϕ*: macrophages; DC: dendritic cells; TNFSF: tumour necrosis factor superfamily; Lck: lymphocyte-specific protein tyrosine kinase; FYN: protooncogene tyrosine-protein kinase Fyn; ITK: IL2-inducible T-cell kinase; PI3K: Phosphoinositide 3-kinase; Grb2: Growth factor receptor-bound protein 2; PI: phosphatidylinositol; PIP3: phosphatidylinositol (3,4,5)-trisphosphate; Akt: PKB is a serine/threonine protein kinase; NF-*κ*B: nuclear factor-kappa-light-chain-enhancer of activated B cells; Sos: son of sevenless homolog 1; Vav1: protooncogene vav; Rac1: Ras-related C3 botulinum toxin substrate 1; JNK: c-Jun N-terminal kinase; CTLA-4: cytotoxic T-lymphocyte-associated protein 4; IL: interleukin; RLK: receptor-like kinase; SHP-2: Src homology region 2 domain-containing phosphatase-2; PP2A: Protein phosphatase 2A; PLC*γ*: Phospholipase C*γ*; B7RP1: B7 related protein 1; ICOS: inducible co-stimulator; ERK: extracellular-signal-regulated kinases; NK-T: natural killer T cells; MEK1/2: mitogen-activated protein kinase kinase 1/2; MAPK: mitogen-activated protein kinase; TNFR1: TNF receptor 1; IFN*γ*: interferon gamma; TRAF: TNF-receptor-associated factors; IkB: inhibitor of *κ*B; Jac3: Janus kinase 3; and STAT5: signal transducers and activators of transcription 5; Ig: immunoglobulin.

*CD80 = B7-1, CD86 = B7-2; **CD70 = CD27L; ^†^OX40 = CD134; ^‡^OX40L = CD134L; and ^€^CD40L = CD154.

**Table 2:** Major translational studies and clinical trials testing various potential co-stimulatory molecules in the treatment of systemic lupus erythematosus.

Molecule (ref.)	Nature	*In vitro*/animal/observational studies in humans	Clinical trial
CTLA-4Ig [37–40, 42, 43, 45]	Recombinant fusion protein	Reduces autoreactive B cells, autoantibodies, IFN*γ* production, and class switching, along with amelioration of GN in animal models Dampens cognate interactions between T-B cells and reduction in autoimmune-driven inflammation	No statistically significant difference in new BILAG A or B flares in a RCT of 175 patients. If only BILAG A was assessed in *post hoc* analyses, more patients on placebo flared than those received Tx (54.4% versus 40.7%). More adverse events were noted in placebo than Tx group (19.8% versus 6.8%) In another RCT of 290 patients with class III or IV lupus nephritis, no significant difference was found between both Tx and placebo groups in complete renal response. Using the ALMS and LUNAR response criteria in *post hoc* analyses, more patients on the Tx group than those on placebo had complete renal response The ACCESS trial is currently underway

Anti-ICOS Ab [76]	mAb	No change in L*ϕ* count and phenotypes in NZB/W F1 mice was noted. Drives production of IFN*γ* & Th2 cytokine and apoptosis upon T-cell stimulation with OVA in SCID mice. In humans, ICOS expression is elevated in CD4+ and CD8+ T cells	Nil

Anti-B7RP1/anti-B7h Ab [76, 78]	mAb	Delays the onset of proteinuria, inhibits IgG production, reduces GN, and prolongs survival in NZB/W F1 mice. Improves renal histology and disease progression in NZB/W F1 mice with established disease	Phase 1b trial (AMG557) for the treatment of stable lupus has just been completed. Data are being awaited

Anti-CD137Ab [64, 65]	mAb	Agonistic to CD137, leading to reduction of GN, splenic CD4+ T cells, anti-dsDNA production, germinal center formation, and reduced mortality in MRL/lpr mice. In NZW/B F1 mice, similar effect as in MRL/lpr mice yet no reduction in splenic CD4+ count but elevation of splenic CD25+ Treg cells In nonlupus human samples, CD137 agonization induces vascular inflammation, plaque formation, and atherosclerosis	Nil

CD134-Fc [72]	Recombinant fusion protein	Reduces Th1 and Th2 cytokine and IFN*γ* production from PBMCs in patients with lupus nephritis	Nil

Anti-CD134Ab [71, 72]	mAb	Reduces IL6, anti-dsDNA and IFN*γ* levels in CD134mAb-treated splenocytes of BXSB mice Reduces Th2 but increases IFN*γ* production in PBMCs of patients with SLE	Nil

Anti-CD40L Ab [60–62]	mAb	Reduces anti-DNA autoantibody production and renal disease and significantly prolongs survival in NZB/W lupus-prone mice. No renal damage and even absence of immune depositions are noted in mice that responded to treatment	A 20-week phase II RCT of 85 patients with mild to moderate SLE receiving IDEC-131 or placebo did not reach both primary and secondary endpoints A phase II open-label trial of 28 patients with proliferative GN receiving BG9588 was terminated prematurely due to 2 cases of cardiac events although significant reduction of proteinuria, haematuria and serum anti-dsDNA titre, and elevation of serum C3 were achieved with BG9588

Abbreviations: ref: references; CTLA-4: cytotoxic T-lymphocyte-associated protein 4; IFN*γ*: interferongamma; GN: glomerulonephritis; BILAG: British Isles Lupus Assessment Group index; RCT: randomized controlled trial; Tx: treatment; mAb: monoclonal antibody; ALMS: Aspreva Lupus Management Study; LUNAR: Lupus Nephritis Assessment with Rituximab; ICOS: inducible costimulator; L*ϕ*: lymphocyte; OVA: ovalbumin; SCID: severe combined immunodeficiency; CD: cluster of differentiation; B7RP1: B7-related protein 1; NZW/B: New Zealand white/black; Treg cells: regulatory T cells; and PBMC: peripheral blood mononuclear cells.
